# A Cross-Sectional, Retrospective, and Comparative Study between Delirium and Non-Delirium Psychiatric Disorders in a Psychogeriatric Inpatient Population Referred to Consultation-Liaison Psychiatry Unit

**DOI:** 10.3390/medicina59040693

**Published:** 2023-03-31

**Authors:** Bernardo J. Barra, Maximiliano Barahona, Luis F. Varela, Pilar Calvo, Anna Bastidas, Jorge Carreño, Luis Pintor

**Affiliations:** 1 Department of Psychiatry, Hospital Clinic i Provincial of Barcelona, University of Barcelona, 08036 Barcelona, Spainlpintor@clinic.cat (L.P.); 2 Mental Health Service, Clínica Universidad de los Andes, Santiago 7591047, Chile; 3 Department of Psychiatry, Medicine School, Universidad Andrés Bello (UNAB), Santiago 8370146, Chile; l.varela@uandresbello.edu; 4 Department of Orthopaedic Surgery, Hospital Clínico Universidad de Chile, Santiago 8380456, Chile; 5 Medicine School, University of Chile, Santiago 8330015, Chile; 6 Department of Psychiatry, Medicine School, Universidad de Santiago de Chile, Santiago 8380456, Chile; 7 Department of Psychiatry, Medicine School, Universidad Mayor, Santiago 8330015, Chile; 8 Institute of Biomedical Research August Pi i Sunyer (IDIBAPS), University of Barcelona, 08036 Barcelona, Spain

**Keywords:** delirium, geriatric psychiatry, aged, inpatient, psychosomatic medicine, consultation-liaison psychiatry

## Abstract

*Background and objectives:* Delirium is the most prevalent psychiatric disorder in inpatient older people. Its presence is associated with higher rates of institutionalization, functional disability and mortality. This study aims to evaluate delirium in a hospitalized psychogeriatric population, focusing on which factors predict the appearance of delirium, the impact it generates and the diagnostic concordance between non-psychiatric physicians and psychiatrists. *Material and methods:* This is an observational, cross-sectional, retrospective, and comparative study. We obtained data from a sample of 1017 patients (≥65 years) admitted to general hospital and referred from different services to the consultation-liaison psychiatry (CLP) unit. Logistic regression was performed using delirium as the dependent variable. To estimate the concordance of the diagnoses, the Kappa coefficient was used. To assess the impact of delirium, an ordinal regression, Wilcoxon median test and Fisher’s test were performed. *Results:* Delirium is associated with a higher number of visits, OR 3.04 (95% CI 2.38–3.88), longer length of stay and mortality, OR 2.07 (95% CI, 1.05 to 4.10). The model to predict delirium shows that being >75 years old has an OR of 2.1 (95% CI, 1.59–2.79), physical disability has an OR of 1.66 (95% CI, 1.25–2.20), history of delirium has an OR of 10.56 (95% CI, 5.26–21.18) and no use of benzodiazepines has an OR of 4.24 (95% CI, 2.92–6.14). The concordance between the referring physician’s psychiatric diagnosis and the psychiatrist CLP unit showed a kappa of 0.30. When analysing depression and delirium, the concordance showed Kappa = 0.46. *Conclusions:* Delirium is a highly prevalent psychiatric disorder, but it is still underdiagnosed, with low diagnostic concordance between non-psychiatric doctors and psychiatrists from CLP units. There are multiple risk factors associated with the appearance of delirium, which must be managed to reduce its appearance.

## 1. Introduction

In recent decades, there has been an increase in the number of older people (aged 65 years or older) admitted to general hospitals [1,2]. These patients present 50–60% of psychiatric comorbidities, a prevalence three to four times higher than those who live in the community [3]. This has generated an increase in hospitalization periods, health costs and morbidity and mortality rates in this group of patients [4,5]. Consultation-liaison psychiatry (CLP) is a subspecialty of psychiatry that concerned with patients with medical and surgical illnesses presenting with psychiatric symptoms in a general hospital [6].

The most prevalent psychiatric disorders in hospitalized older patients are delirium (61%), depression (53%) and dementia (40%) [7]; these three pathologies are known as the 3 Ds of the consultation-liaison psychiatry, which have been associated with high mortality [8,9].

Delirium is an acute and severe neurocognitive disorder [10] characterized by a sudden onset, fluctuating course and disturbances in the level of consciousness that includes alterations in attention, memory, thinking, perception and circadian cycle [11]. Even though delirium is classically described as a reversible condition, elderly patients have poorer outcomes. Its presence worsens the prognosis of the main condition and increases cognitive impairment, the length of hospital stays, institutionalization rates, functional disability, morbidity and mortality [12,13,14]. There are multiple risk factors for developing delirium, such as older age, functional disabilities, male gender, poor vision and hearing, medical and psychiatric pathology, cognitive impairment, laboratory abnormalities and alcohol abuse [15,16]. The diagnosis of delirium is complex and is based on the evaluation of clinical symptoms, physical and neurological examination, laboratory results and measurement tools, such as the confusion assessment method (CAM) which is a widely used standardized instrument designed to allow nonpsychiatric healthcare providers to detect delirium accurately; 4AT is a brief screening tool including four items: alertness, abbreviated Mental Test-4, attention and acute change or fluctuating course. Its score ranges from 0 to 12 points, where a score ≥ 4 suggests possible delirium. Delirium Diagnostic Tool-Provisional (DDT-Pro) is a brief scale designed to allow accurate delirium diagnosis by evaluating vigilance, comprehension and the sleep/awake cycle. The Stanford Proxy Test for Delirium (S-PTD) is fast to administer and is an effective, comprehensive, and simple screening tool for delirium that is robust against fluctuating symptoms and lack of cooperation [17,18,19,20].

The clinical presentation of delirium is variable and is classified as hypoactive, hyper-active and mixed, depending on psychomotor behavior [21]. The hypoactive form occurs more frequently in elderly patients and is often underdiagnosed or misdiagnosed as depression or a form of dementia [22,23].

It is also important to point out, the low concordance rates between the diagnosis of delirium made by the referring physicians of the medical/surgical services and the psychiatrists of the consultation-liaison psychiatry, which are around 30–40% [24]. Among the risk factors for diagnostic discordance, we find the hypoactive subtype of delirium, having a pre-existing psychiatric disorder, the fact that the doctor who refers to the CLP unit is not the treating physician, that is referred from the ICU or a surgical service (services who tend to have a high prevalence of delirium), insomnia and the presence of other central nervous system diagnoses. On the contrary, the factors that would help to achieve higher concordance rates would be the referral from the medical service, elderly patients (>70 years) and the hyperactive subtype of delirium [25,26].

As a result of underdiagnoses of delirium, the inappropriate use of certain psychopharmaceuticals in older people, prolonged hospital stays and high healthcare costs have been observed; therefore, the early detection of delirium is important as it allows for the prevention of associated adverse effects, such as falls, prolonged lengths of hospital stays, cognitive and functional impairment and mortality [27]. Despite its prevalence throughout the lifespan, delirium is a condition that impacts greatly on the elderly, which is why we aimed to study this population.

The objectives of the present study are to (1) evaluate sociodemographic and clinical features of the delirium group compared with other psychiatric disorders, (2) evaluate the impact of delirium in psychogeriatric patients admitted to a general hospital, (3) evaluate which factors predict the appearance of delirium in a hospitalized psychogeriatric population referred to the CLP unit, and (4) evaluate the diagnostic concordance between non-psychiatric physicians and psychiatrists from the CLP unit.

## 2. Materials and Methods

### 2.1. Design

This is an observational, cross-sectional, retrospective and comparative study carried out between 1 January 2016 and 31 December 2018 which gathered all the cases admitted to our unit from 2007 to 2014. The results are reported according to the STROBE statement [28,29].

### 2.2. Patients

**Figure 1:**
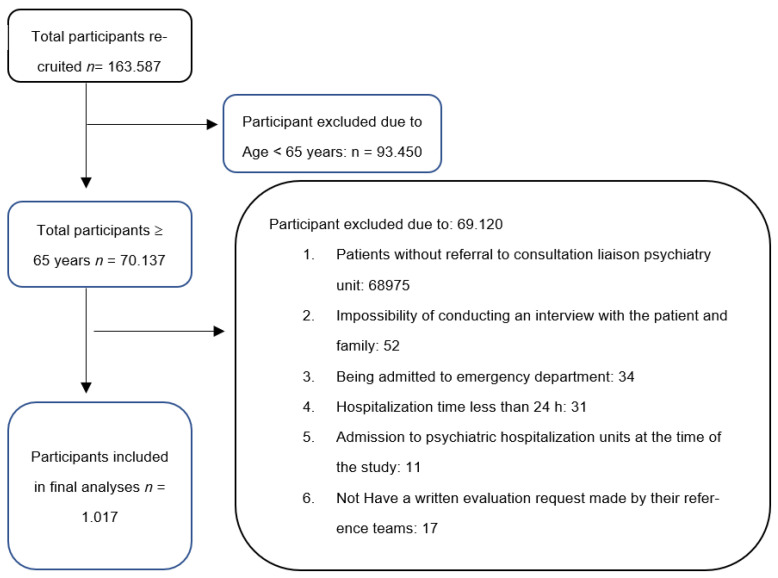
Study flow Diagram.

The participants were from the Clinic Hospital of Barcelona (CHB), which is a tertiary facility that has 819 beds and a catchment area of 540,000 inhabitants within the Barcelona metropolitan area. The total inpatient population admitted at CHB from 2007 to 2014 was n = 163,587; inclusion and exclusion criteria were applied to this sample as shown in Figure 1 (Study Flow Diagram). The final sample for the analysis in this study was n = 1017 participants.

Our study was presented to the Hospital Clinical Research Ethics Committee to obtain their approval to carry out the clinical study (Reg. HCB/20L6/0342 project identification code, date: 4 August 2016). All procedures followed the ethical principles for medical research established in the Declaration of Helsinki [30].

### 2.3. Data Sources and Procedure

The referrals were received by the CLP unit, through the hospital intranet, which delivered:

Sociodemographic variables and clinical characteristics of the sample: age, sex and psychosomatic disorders according to the International Classification of Diseases (ICD-10) [31].

The request made by the department of reference contained the following variables: the date, reference sources (medical specialties), reason for the referral and brief medical history of the patient.

The assessment (anamnesis, diagnosis, treatment, follow-up and data collection obtained from family members, caregivers, referring physicians and bedside nurse) was performed by staff psychiatrists and psychiatrists in training who usually work in our unit.

Referring physicians contributed by making a brief clinical history of the patient, the reason for referral and a brief summary of the daily evolution at each visit by the CLP unit psychiatrist. On the other hand, the bedside nurse contributed by making a brief summary of the daily evolution at each visit by the CLP unit psychiatrist.

To determine the diagnosis of delirium and other psychiatric disorders, we used a clinic interview following the DSM-IV-TR criteria [32].

The data of the patient’s follow-up during the hospital episode, such as psychopharmacological intervention, number of visits, length of hospital stay and destination after discharge were obtained by the psychiatrists and the unit nurse.

The assessment was performed in our Labor Day timetable, which means after the staff meeting that takes place between 9 and 10:30 am. Therefore, patients were evaluated from 11 am to 5 pm, excluding the weekends. All the patients were assessed according to their severity. For instance, delirium patients were evaluated day by day until delirium was remitted. Other Psychiatric disorders were assessed either every day, or every 48–72 h.

All were trained in accordance with European guidelines, and all the cases they evaluated were reviewed by a board-certified faculty psychiatrist [33].

The data obtained were subsequently downloaded to the ACCES software16.0.7 (Microsoft package), where they were stored according to the proposals of the European Consultation/Liaison Workgroup (ECLW) for standardized data collection [34].

### 2.4. Statistical Analyses

We carried out an exploratory analysis; categorical data are presented in absolute and percentage frequencies while discrete continuous data are presented in medians and ranges. Continuous data in which it is acceptable to consider a normal distribution by the Shapiro–Wilk test with a probability >0.15 are presented in means and standard deviations. To compare categorical variables between patients with or without delirium, the Fisher’s exact test or the proportion test was used; meanwhile, for continuous data, the Wilcoxon rank test was used.

A logistic regression analysis was estimated using the diagnosis of delirium as the dependent variable. Those significant variables in the univariate analysis were used to estimate a multivariate model. Discrimination using the receiver operating characteristic (ROC) curve, hat test, and goodness-of-fit test was used to validate the estimated model.

The 95% confidence interval of the area under the ROC curve was estimated, and its discrimination capacity was qualitatively interpreted as suggested by Hosmer and Lemeshow [35]: random: 0.50 to 0.60; low: 0.61 to 0.7; acceptable: 0.71 to 0.80; very good: 0.81 to 0.90; and excellent: 0.91 to 1. In the case of the hat test, a probability <0.05 was accepted as significant for “h” and a probability >0.15 as not significant for “h2” (accept H0).

In the case of the goodness-of-fit test, since it is desirable to accept H0, that is, that the model fits what a binomial distribution predicts, a probability >0.15 was used with a maximum of 10 covariate patterns as acceptable [36].

To estimate the concordance of the diagnoses between the CLP psychiatrist and the referring physician, the Kappa coefficient was used, estimating the 95% confidence interval of the statistic, assuming normal distribution and probability of being different from “0”.

The interpretation of the kappa value was performed using the Landis and Koch classification [37]: poor: 0; mild: 0.01 to 0.20; acceptable: 0.21 to 0.40; moderate: 0.41 to 0.60; considerable: 0.61 to 0.80; and near perfect: 0.81 to 1.

Finally, the impact of the psychiatrist’s diagnosis being delirium compared to another psychiatric diagnosis and whether or not the referring physician agreed with the diagnosis of the liaison psychiatric unit was estimated. The impact was measured in terms of the number of visits, hospital stay, delay in the reference time, type of treatment and mortality. In the first case, an ordinal regression was estimated, using the number of visits grouped as the following as the dependent variable: 1 visit, 2 to 3 visits, 4 to 6 visits and more than 7 visits. The odds ratio and probability of accepting parallelism were estimated with the Brant test, considering a *p* > 0.1 acceptable. In the case of the hospital stay and delay in the reference time, it was compared using the Wilcoxon median test; a *p*-value < 0.05 was considered significant.

In the case of delirium intervention, exploratory analysis and Fisher’s test were performed to compare the impact of the discordant diagnosis.

Finally, for mortality, the OR of dying was estimated, using a univariate model for delirium with respect to another psychiatric diagnosis and a model for the case of discordant diagnoses.

## 3. Results

On average, the patients in the sample were 75.73 ± 6.5 years old and ranged from 66 to 98 years of age. The percentage by gender of the sample was: 50.54% female. The sociodemographic and clinical features of the delirium group compared with other psychiatric disorders are shown in Table 1.

### 3.1. Delirium Impact

#### 3.1.1. Number of Visits

The diagnosis of delirium is associated with an increase in the number of visits (OR 3.04 (95%CI 2.38–3.88)); however, the parallelism test to validate the model is borderline (*p* = 0.111). The number of visits in those patients with a discrepancy in the diagnosis of delirium referral did not significantly increase the number of visits (OR 1.27 (95%CI 0.89–1.83)). See Table 2.

#### 3.1.2. Length of Stay

The length of the hospital stay in patients with a diagnosis of delirium made by a psychiatrist was 20 days (interquartile range: 11 to 40 days), while the hospital stay in patients with another psychiatric diagnosis was 13 days (interquartile range: 8 to 27 days) (*p* < 0.000).

In those patients with a diagnosis of delirium concordant with the liaison psychiatrist and the referring physician, the hospital stay is 18 days (range, 2 to 761). In cases where there is a discrepancy, the hospital stay is 30 days (range: 1 to 194) (*p* = 0.0074).

#### 3.1.3. Delay in Making the Referral to the CLP Unit

Referral for delirium to the CLP unit has a median in terms of delay of 7 days (interquartile range: 3 to 17 days), and in case of other psychiatric diagnoses, it is 6 days (interquartile range: 3 to 12 days) (*p* = 0.0019).

In those patients with a diagnosis of delirium made by the CLP unit, the delay in making the referral when there is concordance in the diagnosis is 6 days (range, 2 to 747); when there is a discrepancy, the median is 9 days (range: 0 to 142) (*p* = 0.0074).

#### 3.1.4. Treatment

The differences in the therapeutic management of delirium compared to other psychiatric pathologies (non-delirium) are shown in Table 3.

The use of antipsychotics was significantly higher in patients with a diagnosis of delirium, as was the use of antidepressants in patients with a psychiatric diagnosis other than delirium; these differences were statistically significant.

In those patients where there was a discrepancy with the diagnosis of the referral unit, the need to use antipsychotics was more frequent (n = 218, 81.3%) compared to those patients where there was concordance between the referral service and the CLP unit (n = 122, 68.9%) (*p* = 0.003).

#### 3.1.5. Mortality

The number of deaths was 36 patients; 22 (61.1%) of them were diagnosed with delirium by the CLP unit, while 14 were diagnosed with another psychiatric diagnosis (38.9%). The OR of dying having been diagnosed with delirium by the CLP unit is 2.07 (95%CI, 1.05 to 4.10).

The error in the referral service diagnosis did not significantly increase the risk of dying from delirium, with the mortality being 4.52% in those patients in whom the referral service and CLP unit agreed and 5.22% in cases when the CLP unit diagnosed delirium and the referral service diagnosed another diagnosis (OR 0.86 (95%CI, 0.35–2.09)).

### 3.2. Predictors of Delirium

In the univariate analysis, the following were significant risk variables for the diagnosis of delirium: patient age (OR 1.08 (95%CI 1.06–1.10)), physical disability (OR 1.86 (95%CI 1.45–2.39)), having a medical history of delirium (OR 12.32 (95%CI 6.30–24.08)), no other psychiatric history (OR 1.53 (95%CI 1.19–1.96)), and no environmental stressors (OR 1.64 (95%CI 1.22–2.22)). On the other hand, alcohol consumption (OR 0.09 (95%CI 0.04–0.21)) and benzodiazepine use (OR 0.25 (95%CI 0.18–0.35)) were protective factors for the diagnosis of delirium given that they were referred to the CLP unit.

The multivariate model to predict the diagnosis of delirium by the CLP unit psychiatrist included the following variables: age, physical disability, delirium medical history, no environmental stressors and benzodiazepine use. This model presented acceptable discrimination with an area under the ROC curve of 0.75 (95%CI, 0.72 to 0.78).

The goodness-of-fit test presented 28 covariates and a probability of 0.5804. The hat test showed a significant *h* (*p* < 0.000) and a probability of 0.896 in *h2*. Both the goodness-of-fit test and the hat test confirm the assumptions of the model.

The multivariate model shows that being >75 years old has an OR of 2.1 (95%CI, 1.59–2.79); physical disability has an OR of 1.66 (95%CI, 1.25–2.20); delirium medical history has an OR of 10.56 (95%CI, 5.26–21.18); no environmental stressors has an OR of 1.91 (95%CI, 1.36–2.67); and no benzodiazepine use has an OR of 4.24 (95%CI, 2.92–6.14). See Table 4.

### 3.3. Diagnostic Concordance

The percentage of agreement between the diagnosis of the referring physician and the CLP unit psychiatrist was 43.56%, obtaining a kappa of 0.30 (95%CI, 0.27–0.32), *p* < 0.000, i.e., acceptable concordance.

In the case in which the psychiatrist’s diagnosis was delirium, the percentage of agreement was 79.35%, obtaining a kappa of 0.56 (95%CI, 0.51–0.62), *p* < 0.000, i.e., a moderate concordance. When analyzing the cases with depression and delirium in detail, the percentage of concordance between the referring physician and CLP unit psychiatrist was 76.54%, with a moderate concordance (kappa = 0.46, 95% CI 0.38 to 0.54). See Table 5.

## 4. Discussion

The main finding of our study was the confirmation that there are risk factors for developing delirium in geriatric patients. These factors are listed in descending order: a medical history of delirium, advanced age (especially persons >75 years of age) and physical disability. These findings are to be expected, are concordant with what has been published in the literature and are related to the higher risk of developing delirium in patients who have previously presented it. One of the possible reasons is the cognitive impairment presented by post-delirium patients, which in some cases could lead to dementia, as indicated by the meta-analysis carried out by Pereira et al., who have shown that delirium increases the chances of developing dementia by approximately twelve times (OR = 11.9 [95% CI 7.3–19.6], *p* < 0.001), strongly emphasizing that delirium is a significant risk factor for incident dementia.

Although the exact pathophysiological mechanism linking them is not yet known, it is suggested that delirium could act as the acute exacerbation of dementia, with acute episodes driving the onset and progression of the underlying chronic disease (dementia). The onset of post-delirium dementia may be due to factors such as delirium subtype, severity, duration, stroke, and/or psychiatric illness. The appearance of delirium should generate concern in physicians in order to prevent future episodes and to avoid further cognitive impairment that could generate dementia in the same patient in the future [38].

Physical disability has been widely associated as a risk factor for the onset of delirium; an example of this is the study by Sidoli et al. [39] in which 1237 patients aged 65 years or older were studied and it was found that non-modifiable factors, such as physical disability, and modifiable factors, such as physical restrictions, were associated with the onset of delirium.

Similarly, the study by Wilson et al. [40] points out that physical disability is part of the geriatric syndrome frailty, which has been widely associated with the onset of delirium in the elderly. With respect to advanced age, the literature [41] associates it with an increased risk of developing delirium, which is more complicated and has a worse prognosis [42].

Another interesting finding in our study is that the consumption of alcohol and the use of benzodiazepines would act as protective factors against the onset of delirium, results that contradict the literature, which points them out as risk factors for the onset of delirium due to the sedative effects that affect the central nervous system [43].

Although our finding can be interpreted as counterintuitive, it forces us to think of new possibilities. In this study, we consider only those who were referred to the consultation-liaison psychiatry unit.

Since the diagnosis of delirium is the most prevalent psychiatric condition in the general hospital, it is possible that most symptoms of confusion or delirium are managed by their treating teams, so there could be particularities in patients with delirium who are referred to the CLP unit. The specific motor subtype or other psychopathological characteristics were not included in this study. It is important to consider that different series have shown the high prevalence of catatonic symptomatology in patients with delirium, where benzodiazepines could have a therapeutic role.

The use of benzodiazepines continues to be a precipitant or perpetuator of delirium, except when the use of benzodiazepines can directly influence the pathophysiology to be treated, such as GABAergic withdrawal syndrome.

Regarding the impact of delirium in our sample, the findings were as follows:

When evaluating the number of visits made by the CLP unit, the greater need for these visits in patients with delirium compared to those with other psychiatric diagnoses stands out. This is concordant with what is described by Navinés et al. [44], that patients with a diagnosis of delirium required at least one more visit by CLP unit psychiatrists than those with other psychiatric diagnoses.

This could be related to the greater clinical complexity of delirium, the error at the time of diagnosis, associated with a worsening of the condition, and a longer delay in referral to the CLP unit. This greater use of hospital services would be closely related to the increase in health costs widely described in the literature.

Regarding the length of stay, we found that patients with a diagnosis of delirium had a longer hospital stay than those with other psychiatric diagnoses. Our results coincide with those described in the study by Kirfel et al. [45], in which those patients with delirium had a longer hospital stay (26.5 ± 26.1 days) than those without delirium (14.6% ± 6.7 days). In addition, it showed that delirium was an independent predictor of prolonged length of stay (LOS).

Another more recent study by Kirfel et al. [46] obtained similar results, showing a significant difference in total LOS in the hospital of approximately 8 days. Patients who developed delirium stayed approximately 26 days (25.6 ± 17.2) and patients without delirium stayed a mean of 17 days (17.2 ± 25.7; *p* < 0.001).

This generates an increase in social costs (the need for post-acute care and demand for unpaid caregivers), healthcare and, in a significant number of cases, loss of functionality or neurocognitive impairment in the elderly [47,48,49].

Regarding the delay in the referral of patients with delirium from the different medical and surgical services to the CLP unit, it was found that the longest delay is generated when there is a discrepancy in the psychiatric diagnosis between a non-psychiatrist physician who makes the referral and the CLP unit psychiatrist. These results agree with those described by Grover et al. [50], wherein non-psychiatrist physicians do not identify delirium early in several situations and this generates a delay in referral to psychiatry. The average referral time to the CLP unit is 3.0–5.3 days, but the range can vary from 1 to 40.

Among the factors that were associated with a greater delay in referral to the CLP unit were: age (the older the patient, the greater the delay), the hypoactive subtype of delirium, the absence of previous psychiatric history, admission to an ICU and sleep–wake cycle disorders, sometimes considered by physicians as a normal phenomenon in hospitalizations.

Concerning the management received by our patients, the most used was pharmacological, antipsychotics being the most frequent, followed by non-pharmacological management. Although we know that the treatment of delirium is the improvement of the underlying medical cause, the use of antipsychotic drugs has been widely used for the management of symptoms such as hallucinations, delusional ideas and psychomotor agitation.

Even though the evidence on their use and efficacy is still contradictory [51] and the studies conducted have multiple limitations and heterogeneous results, they are not convincing to apply their use in any hospital setting. A systematic review published by the Cochrane Database [52] showed that antipsychotics did not reduce the severity or resolve the symptoms of delirium when compared with other drugs, and they have even been associated with an increased risk of cardiac (QT interval prolongation, cardiac arrhythmias, etc.) or cerebrovascular events, even when used in the short term. In addition, the FDA has not approved the use of these drugs for the treatment of delirium. Despite the above, studies show a high use of antipsychotics in patients with delirium (77–87%). In relation to the above, it seems important to us to make rational, reflexive use of these drugs, limited to specific objectives such as psychotic symptoms and agitation [53].

In the case of having to use them, risperidone, olanzapine and quetiapine would have greater support in the literature, an example of which is the positive results obtained when treating delirium with quetiapine in ICU settings [54].

In addition, the use of multicomponent non-pharmacological measures for the prevention and management of delirium should be highlighted, such as orientation, early ambulation, normalization of the sleep–wake cycle, the use of devices (e.g., glasses or hearing aids), hydration, etc., which have been extensively studied in recent years and have shown moderate certainty of evidence in the improvement of delirium [55].

The greater use of antipsychotic drugs, as there is a discrepancy in the diagnosis of the reference unit, would be related to the greater number of delirium cases diagnosed by the CLP unit.

We believe it is important to highlight the association with a higher risk of death in those patients with a diagnosis of delirium versus those with another psychiatric diagnosis. This relationship is consistent with that described in the review by Tachibana et al. [56], wherein delirium was associated with an increased risk of mortality during hospitalization and even after discharge, acting as an independent risk factor. In the same review, delirium was associated with an increase in perioperative mortality (30-day in-hospital mortality: RR: 2.79, 95% CI: 1.97–3.93). Similarly, the study by Park et al. [57] observed a significant association between the presence of delirium and mortality in the elderly, both in hospital (OR = 3.34, CI = 1.21–9.19) and at 6 months after discharge (HR = 2.85, CI 1.28–6.36). Therefore, prevention, early detection and adequate management of delirium are essential.

Regarding the diagnostic concordance between non-psychiatrists and CLP unit psychiatrists, it seems important to note that the referral rate to the CLP unit for delirium and depression is high, which is similar to the high incidence of both psychiatric pathologies in hospitalized elderly people [58,59,60].

After evaluation by the CLP unit, the diagnosis of delirium was even higher with 43.76% of cases, which is consistent with that described in the literature by Fuchs et al. [61], who point out that the prevalence of delirium in patients older than 65 years is within the range of 11–50% during their hospitalization. Despite the high prevalence, it is often underdiagnosed or misdiagnosed [62,63] in up to 70% of cases [64].

It is important to keep in mind that delirium is a common and reversible disorder in hospitalized elderly people, and its early diagnosis may decrease care costs, increase nursing home discharges, and alleviate long-term cognitive impairment [65,66,67].

When analyzing the concordance between the psychiatric diagnoses made by the referring physician and the CLP unit team, 43.56% agreement was obtained, a value similar to the 41.5% obtained in the study by Su et al. [68] but below that described by Wancata et al. [69].

In the latter, agreement reached 50% of diagnoses made correctly to hospitalized patients presenting diagnosed psychiatric symptoms. The kappa in our sample was 0.30, which is only considered acceptable. This low diagnostic accuracy shows the described risk that non-psychiatric physicians may not easily recognize or misdiagnose psychiatric disorders.

This diagnostic discrepancy could be due to multiple factors, such as the patient’s own factors (psychological state at the time of the interview, atypical presentations of psychiatric disorders, indirect information, etc.) and medical factors (unstructured interviews, previous training, work experience, etc.), as noted in the study by Otani et al. [70]. Other factors could be related to a high workload and short attention time to evaluate the patient [71].

To decrease diagnostic discrepancy, it would be necessary for unit CLP teams to provide support through proactive rather than reactive patient search, ongoing training of general hospital staff and a reduction in the stigma of inpatient mental health problems [72,73,74].

Another finding that we observed in the sample is that the agreement between the referring physician and psychiatrists is moderate (kappa 0.46) when talking about delirium and depression, which is consistent with the findings of Yamada et al. [75].

This finding could be due to the training provided by the CLP unit to the medical teams of our hospital, which would have allowed them to develop clinical skills for the correct recognition of psychiatric syndromes. Due to the above, we believe it is essential to continue actively training the different medical teams to further reduce the gaps in knowledge and early detection of complex psychiatric syndromes.

### Limitations

There are limitations in this study: it is retrospective, cross-sectional, naturalistic and of clinical practice, which can generate more inaccuracies without a protocolized follow-up of a fixed number of visits. Another limitation is the absence of quantitative data specific to the context of delirium (e.g., severity of delirium; type, duration and number of occurrences per patient), screening instruments such as scales were not used to make psychiatric diagnoses. The study was conducted in a single tertiary-level center (university hospital in an urban area), which does not allow the results to be generalized.

Patient follow-up was performed only during hospitalization. The sample was selected by physicians, not psychiatrists; it was not an active search for all the hospitalized elderly, which would have given us a less biased sample. We have not considered aspects of the medical pathology or the pharmacological approach beyond psychoactive drugs. Another limitation of our study is that the dementia variable was not included, excluding a known predictive risk factor for the appearance of delirium in the elderly population, which generates a bias in our study.

A strength of our study is the size of the sample (1017 patients), which is focused exclusively on the geriatric population. We were able to assess how often delirium is reported and what its usual care resembles in a regular hospital setting.

## 5. Conclusions

Delirium is a highly prevalent psychiatric disorder in hospitalized older people; despite this, it is still underdiagnosed by medical and surgical teams, which is observed in the low diagnostic concordance between non-psychiatric physicians and CLP unit psychiatrists.

This is serious, as delirium is associated with higher mortality, longer referral times to the psychiatry unit consultation-liaison, greater demand from the medical team, prolonged length of hospital stay, and a high use of antipsychotics. There are multiple risk factors associated with the appearance of delirium, which must be addressed and managed by the medical team to reduce its appearance.

CLP units will need to place more emphasis on older people and have professionals trained in geriatric psychiatry to address the needs of this group; in addition, they should be conducting continuing education for non-psychiatric physicians on the diagnosis and management of delirium. We believe it is necessary to carry out randomized clinical trials which will allow us to delve into the impact of delirium and risk factors in the elderly. These additional studies will facilitate a better understanding of the clinical profiles of elderly patients, which would allow us to carry out better management.

## Figures and Tables

**Table 1:** Sociodemographic and clinical features of delirium group compared with the others psychiatric disorders.

	Delirium N = 445 (43.76%)	No Delirium N = 572 (56.24%)	Total N = 1017 (100%)	*p*-Value
Age (years)	77	74	75	<0.000 (w)
Gender				
- Female	222 (49.89%)	292 (51.05%)	514 (50.54%)	NS (f)
- Male	223 (50.11%)	280 (48.95%)	503 (49.46%)	
History of Delirium	80 (17.98%)	10 (1.75%)	90 (8.84%)	<0.000 (f)
History of Substance Dependence	16 (3.60%)	76 (13.29%)	92 (9.04%)	<0.000 (f)
History of Alcohol Dependence	6 (1.35%)	76 (13.29%)	82 (8.06%)	<0.000 (f)
Psychiatric Diagnosis Referring Service				
Anxiety Disorder	21 (4.72%)	77 (13.50%)	98 (9.64%)	<0.000 (f)
Depression	101 (22.70%)	264 (46.15%)	365 (35.89%)	<0.000 (f)
Delirium	268 (60.22%)	33 (5.77%)	301 (29.60%)	<0.000 (f)
Substance Dependence	1 (0.22%)	68 (11.89%)	69 (6.78%)	<0.000 (f)
Psychosis	13 (2.92%)	17 (2.97%)	30 (2.95%)	NS (f)
Personality Disorder	3 (0.67%)	9 (1.57%)	12 (1.18%)	NS (f)
Adaptive Disorder	23 (5.17%)	68 (11.89%)	91 (8.95%)	<0.000 (f)
Suicide Attempt	6 (1.35%)	28 (4.90%)	34 (3.34%)	0.001 (f)
Others	9 (2.03%)	8 (1.40%)	17 (1.67%)	NS (f)
Referring Service				
General Medicine	105 (23.60%)	192 (33.58%)	297 (29.20%)	NS (p)
Medical Subspecialties ^†^	121 (27.19%)	187 (32.69%)	308 (30.29%)	NS (p)
Surgery ^‡^	179 (40.22%)	149 (26.04%)	328 (32.25%)	0.0069 (p)
Neurology	40 (8.99%)	44 (7.69%)	84 (8.26%)	NS (p)
Environmental Stressors				
Economics	9 (2.02%)	25 (4.37%)	34 (3.34%)	NS (f)
Familiar	73 (16.42%)	131 (22.90%)	204 (20.06%)	NS (f)
Judicial	1 (0.22%)	3 (0.53%)	4 (0.39%)	NS (f)
Physical Abuse	1 (0.22%)	0 (0%)	1 (0.10%)	NS (f)
Problem at work	1 (0.22%)	1 (0.17%)	2 (0.20%)	NS (f)
No Environmental Stressors	360 (80.90%)	412 (72.03%)	772 (75.91%)	0.001 (f)
Physical Disability				
- Autonomous	192 (3.15%)	335 (58.57%)	527 (51.82%)	<0.000 (f)
- Needs Assistance	253 (56.85%)	237 (41.43%)	490 (48.18%)	
Discharge Disposition				
Nursing Home	63 (14.16%)	69 (12.06%)	132 (12.98%)	
Home	352 (9.10%)	492 (4.27%)	834 (82.01%)	
Death	22 (4.94%)	14 (2.45%)	34 (3.44%)	
Others	8 (1.80%)	7 (1.22%)	15 (1.48%)	NS (f)

Abbreviations: NS = not significant. N, number (frequency); %, percentage been calculated on subtotals; † includes: cardiology, hematology/oncology, nephrology/urology services; ‡ includes: surgery and trauma services. Note: *p* values were calculated using: (f) = Fisher’s exact test; (P) = test for proportions; (w) = Wilcoxon rank test. Statistically significant difference: *p* < 0.01. Environmental stressors understood as a set of variables that are perceived as aversive for the person and that could influence the probability of developing delirium.

**Table 2:** Comparison in the number of visits made by psychiatrists from the consultation-liaison psychiatry (CLP) unit to patients with delirium versus other psychiatric disorders.

Nº Visits	Delirium N = 445	No Delirium N = 572	Total N = 1017	*p*-Value
1 visit	94 (21.12%)	241 (42.13%)	335 (32.94%)	<0.000 (f)
2–3 visits	220 (49.44%)	270 (47.21%)	490 (48.18%)	
4–7 visits	98 (22.02%)	54 (9.44%)	152 (14.95%)	
>7 visits	33 (7.42%)	7 (1.22%)	40 (3.93%)	

Abbreviations: N, number (frequency); %, percentage been calculated on subtotals; CLP, consultation-liaison psychiatry. Statistical test used: (f) = Fisher’s exact test. Statistically significant difference: *p* < 0.01.

**Table 3:** Treatment of patients with delirium and patients with another psychiatric diagnosis (non-delirium) by CLP units.

Pharmacological Prescription by CLP Unit	Delirium N = 445	No Delirium N = 572	Total N = 1017	*p*-Value
Antidepressants	29 (6.5%)	269 (47.0%)	298 (29.30%)	* *p* < 0.001
Antipsychotics	340 (76.4%)	89 (15.6%)	429 (42.18%)	* *p* < 0.001
Mood Stabilizer	6 (1.3%)	13 (2.3%)	19 (1.87%)	NS
Benzodiazepines	7 (1.6%)	94 (16.4%)	101 (9.93%)	NS
No Prescription	63 (14.2%)	107 (18.7%)	170 (16.72%)	NS

Abbreviations: N, number (frequency); %, percentage been calculated on subtotals; NS, not significant; CLP, consultation-liaison psychiatry. Note: *p*-values were calculated using the Pearson’s chi-squared test. * *p* < 0.01.

**Table 4:** Predictors of delirium according to the patient’s profile.

			No Delirium History		Delirium History	
Age (Years)	Physical Disability	BZD	No Environmental Stressor	Environmental Stressor	No Environmental Stressor	Environmental Stressor
**<75 Years**	No Physical Disability	No BZD	0.34 (0.29–0.40)	0.22 (0.15–0.28)	0.85 (0.75–0.94)	0.74 (0.60–0.89)
		BZD use	0.11 (0.07–0.15)	0.06 (0.03–0.09)	0.57 (0.38–0.76)	0.41 (0.21–0.61)
	Physical Disability	No BZD	0.47 (0.39–0.54)	0.31 (0.24–0.39)	0.90 (0.84–0.97)	0.83 (0.72–0.93)
		BZD use	0.17 (0.11–0.23)	0.10 (0.06–0.14)	0.68 (0.52–0.85)	0.53 (0.34–0.73)
**>75 Years**	No Physical Disability	No BZD	0.47 (0.41–0.54)	0.37 (0.28–0.45)	0.92 (0.87–0.97)	0.86 (0.77–0.95)
		BZD use	0.21 (0.14–0.27)	0.12 (0.07–0.17)	0.73 (0.58–0.88)	0.59 (0.39–0.79)
	Physical Disability	No BZD	0.65 (0.59–0.71)	0.49 (0.41–0.57)	0.95 (0.92–0.98)	0.91 (0.85–0.97)
		BZD use	0.30 (0.22–0.38)	0.19 (0.12–0.25)	0.82 (0.71–0.93)	0.71 (0.54–0.87)

Abbreviations: BZD: benzodiazepine.

**Table 5:** Diagnostic accuracy between non-psychiatric physicians and CLP unit.

Non-Psychiatric Physicians	Psychiatric		Total
	Depression	Delirium	
**Depression**	81	101	182
**Delirium**	6	268	274
**Total**	87	369	456

## Data Availability

Not applicable.
